# Exosomal miR-125b-5p derived from mesenchymal stromal/stem cell enhances anti-PD-1 therapy in mouse colon cancer model

**DOI:** 10.1186/s13287-025-04227-3

**Published:** 2025-03-05

**Authors:** Mengmeng Jiang, Jia Liu, Shengquan Hu, Xueqin Yan, Yongkai Cao, Zhengzhi Wu

**Affiliations:** 1 Shenzhen Institute of Translational Medicine, The First Affiliated Hospital of Shenzhen University, Shenzhen Second People’s Hospital, Shenzhen, 518035 China; 2 Department of Neurology, The First Affiliated Hospital of Shenzhen University, Shenzhen Second People’s Hospital, Shenzhen, 518035 China; 3 Department of Pharmacy, Changchun University of Chinese Medicine, Changchun, Jilin 130117 China; 4 Wu Zhengzhi Academician Workstation, Ningbo College of Health Sciences, Ningbo, 315800 China

**Keywords:** Exosome, Mesenchymal stromal/stem cell (MSC), miR-125b-5p, Anti-PD-1 therapy, Tregs, Colon cancer

## Abstract

**Background:**

There is compelling evidence that FoxP3^+^ regulatory T cells (Tregs) play a critical role in promoting tumor immune evasion. Our prior research demonstrated that the expression of miR-125b-5p directly inhibits Tregs by targeting TNFR2 and FoxP3. Given the significant therapeutic potential of mesenchymal stromal/stem cell (MSC)-derived exosomes (MSC-EXO) in cancer treatment, the potential role of MSC-EXO in augmenting anti-tumor immunotherapy through the delivery of miR-125b-5p remains unexplored.

**Methods:**

Nanoparticle tracking analysis (NTA) and transmission electron microscopy (TEM) were employed to characterize exosomes derived from MSCs. Flow cytometry analysis was conducted to investigate the function of exosomal miR-125b-5p both in vitro and in vivo. Mouse MC38 tumor models were administrated MSC-derived exosomes containing miR-125b-5p via tail vein injection, with or without the concurrent injection (intraperitoneally, i.p.) of anti-PD-1 antibodies.

**Results:**

Our results indicated that exosomal miR-125b-5p derived from MSC significantly inhibited the expansion, proliferation and suppressive function of Tregs in vitro. Moreover, we observed a marked reduction in tumor growth in mice treated with exosomal miR-125b-5p. Notably, while anti-PD-1 therapy alone achieved a cure rate of approximately 30% in a mouse model of colon cancer, the combined administration of exosomal miR-125b-5p significantly enhanced the therapeutic efficacy, resulting in a more than two- to three-fold increase in tumor regression in approximately 80% of the treated mice. The underlying cellular mechanism was closely associated with the reduction of tumor-infiltrating Tregs. and the increase of CD8^+^ cytotoxic T lymphocytes (CTLs).

**Conclusions:**

In summary, our findings suggest that exosomal miR-125b-5p derived from MSC exerts prominent potential in advancing anti-PD-1 therapy by modulating tumor immune environment. This property of miR-125b-5p may be therapeutically harnessed in human cancers to enhance the efficacy of immunotherapy.

**Graphical Abstract:**

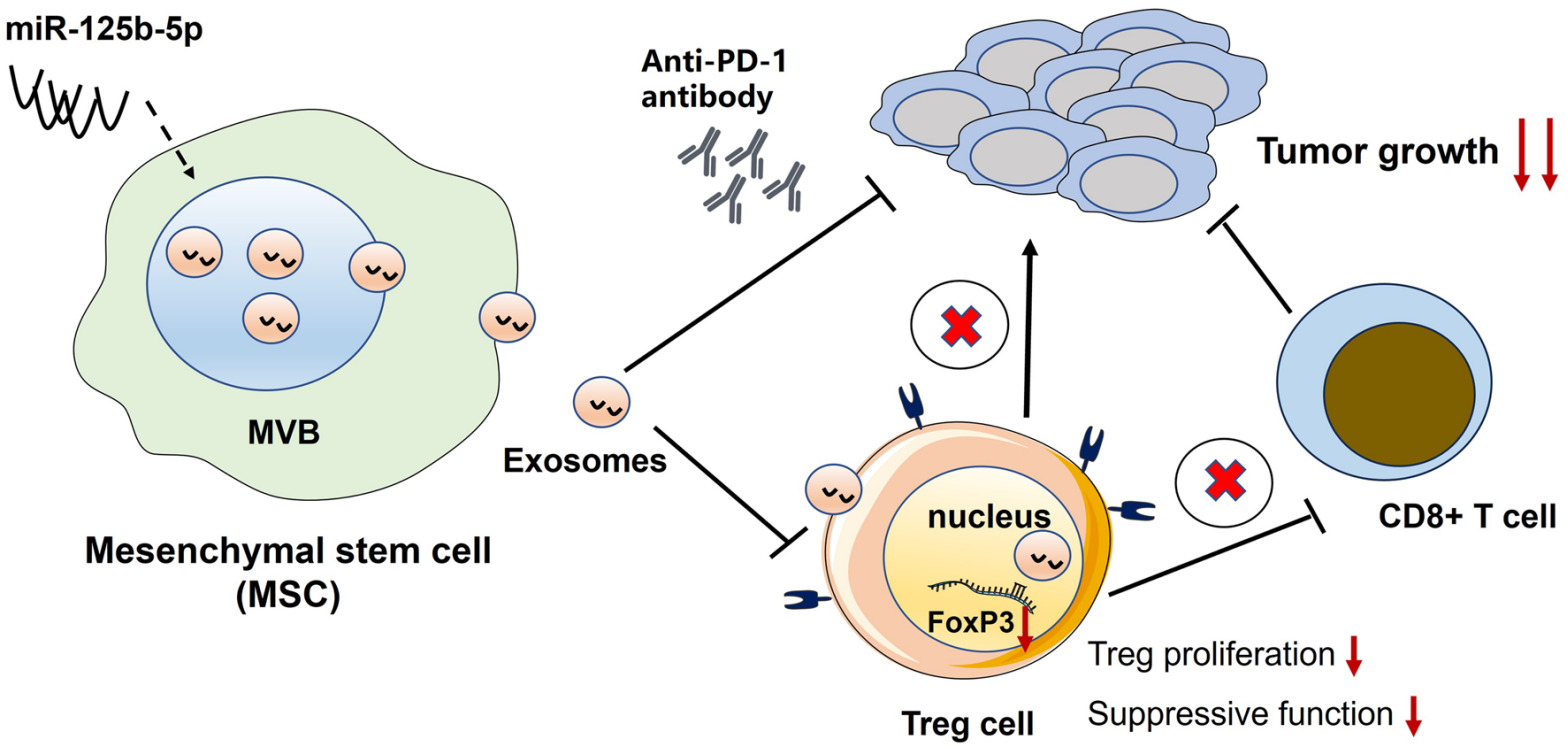

**Supplementary Information:**

The online version contains supplementary material available at 10.1186/s13287-025-04227-3.

## Introduction

Immunotherapy has revolutionized the treatment of human cancers, with immune checkpoint inhibitors (ICIs) such as anti-PD-1/PD-L1 and anti-CTLA4 therapies being extensively utilized in clinical settings for various cancer patients. However, the overall complete response (CR) rate for these treatments remains limited to approximately 20~30% [1]. Accumulating evidence suggests that the presence of abundant CD4^+^FoxP3^+^ regulatory T cells (Tregs) within tumor tissue significantly contributes to the limited efficacy of ICIs in cancer treatment [2]. Thus, targeting Tregs may greatly enhance the therapeutic efficacy of cancer immunotherapy. Recent studies have demonstrated that TNFR2 is preferentially expressed by highly suppressive Tregs and certain tumor cells. The accumulation of TNFR2-expressing Tregs in cancer patients is correlated with more severe clinicopathological features and poorer prognoses [3, 4]. Notably, our analysis of single-cell RNA sequencing data from various human cancer types, suggests that TNFR2 expression levels are closely associated with the response to ICIs treatment [5]. Therefore, modulating the stability of TNFR2^+^ Tregs may be significant in enhancing anti-tumor immunotherapy.

In our previous research, we first found and reported that TNFR2 could be downregulated directly by miR-125b-5p in Tregs [6], and meanwhile, in vivo treatment with miR-125b-5p agomir (chemical agonist) potently inhibited the tumor growth in mouse colon tumor models. It has been well established that miR-125b-5p is a tumor suppressor with the capacity to suppress various tumor types [7]. We thus hypothesize that the efficient delivery of active miR-125b-5p in tumor environment may enhance the efficacy of ICIs in cancer treatment. Mesenchymal stromal/stem cells (MSCs) are increasingly utilized in cell therapy due to their potent immunomodulatory and regenerative abilities, primarily through their paracrine activity. Recent studies highlight the extracellular vesicles, particularly exosomes, play a key role in enhancing the therapeutic efficacy of MSCs [8]. Currently, exosomes are recognized as highly effective delivery vectors for numerous microRNAs [9]. Nevertheless, MSC-derived exosomes (MSC-EXO) exhibit a notable capacity to target tumor tissues and exert substantial anti-tumor effects by modulating the tumor immune microenvironment or directly targeting tumor cells. Given that bone marrow (BM)-derived MSC exhibits a more stable capacity for proliferation and differentiation, which facilitates their manipulability and investigational potential, BM-derived MSC are being recognized as a promising therapeutic option [10]. The recognition is supported by a growing number of clinical studies exploring their effects.

To test whether exosomal miR-125b-5p derived from MSC could enhance the therapeutic efficacy of anti-PD-1 treatment in colon cancer, we first generated miR-125b-5p-overexpressing MSCs and purified their exosomes. Intriguingly, we found that the exosomes expressing miR-125b-5p derived from MSC (MSC-EXO-miR-125b-5p) significantly inhibited the expansion, proliferation, and suppressive function of FoxP3^+^ Tregs. Moreover, the anti-tumor efficacy of anti-PD-1 therapy was substantially enhanced in mice concurrently administered with exosomal miR-125b-5p. Thus, we believe that MSC-derived exosomal miR-125b-5p could be used as a novel therapeutic strategy for enhancing ICIs therapy in cancer treatment. The results of this study provide basically evidence that miR-125b-5p delivered by exosomes may be targeted to enhance the outcomes of immunotherapy of cancer patients in clinic.

## Materials and methods

### Mouse, cell and reagents

Female C57BL/6J mice (wild-type, 8–12 weeks old) were provided and maintained by the Animal Facility of Changchun University of Chinese Medicine. The mouse colon cancer cell lines of MC38 were purchased from ATCC. The mesenchymal stromal/ stem cell (MSC) from mouse bone marrow which has been utilized widely [11, 12], were purchased from Wuhan Pricella Biotechnology Co., Ltd. (Cat# CP-M131). The flow cytometry fluorescently conjugated antibodies of FITC anti-mouse CD45 (clone: 30-F11), PerCP-Cy5.5 anti-mouse TCRβ (clone: H57-597), and PE-Cyanine7 anti-mouse CD4 (clone: GK1.5) were purchased from eBioscience; PE anti-mouse CD120b/TNFR2 (clone: TR75-89), and APC anti-mouse FoxP3 (clone: FJK-16s) were purchased from Invitrogen. PE anti-mouse IFN-γ (clone: XMG1.2), and APC anti-mouse CD8a (clone: 53 − 6.7) were obtained from Biolegend. Recombinant mouse IL-2 (200 µg/ml) and TNF (200 µg/ml) were purchased from BD Pharmingen. *InVivoMAb* anti-mouse PD-1 (CD279) antibody (Clone RMP1-14, Cat# BE0146) and its isotype control (Armenian hamster IgG) were purchased from Bio X Cell.

### Cell culture and miRNA transfection

The mouse MSC and MC38 cells were both cultured in Dulbecco’s modified medium (DMEM, Gibco), which contained 10% exosome-depleted FBS, growth factors, and 1% penicillin/streptomycin (100×), at 37℃, in a 5% CO_2_ atmosphere. The lentiviral vector expressing miR-125b-5p (LV-miR-125b-5p) and its empty vector control (LV-miR-NC) were generated by Genechem (Shanghai, China). For the stable expression of miR-125b-5p in MSC cells, GFP labeled LV-miR-125b-5p was infected into MSC cells and selected by 5 µg/ml puromycin for 7~10 days. All experimental protocols were conducted according to the manufacturer’s instructions.

### Exosome isolation and characterization

The exosomes were isolated and purified from MSC cells (WT, LV-miR-NC, LV-miR-125b-5p) according to the following procedures. Firstly, the cell culture supernatants were centrifuged at 2,000 g for 10 min to remove cells and 10,000 g for 30 min to deplete the residual cell debris. Afterward, samples were condensed to 3 ml~5 ml through Ultrafiltration Spin Columns (10 kDa, Millipore), and the concentrated supernatants were used to precipitate exosomes by using ultracentrifugation at 100,000 g for 2 h. The size and morphology of isolated exosomes were identified by nanoparticle tracking analysis (NTA), transmission electron microscopy (TEM) as described previously [13].

### RNA isolation and miRNA analysis

QIAzol-based total RNA purification from MSC-derived exosomes was performed by using RNeasy Mini Kit (Qiagen, Cat# 74104), and 0.5~1µg of total RNA was transcribed into cDNA with specific miRNA RT primers. The Bulge-loop™ miRNA RT-qPCR primer sets for the quantification of miR-125b-5p were synthesized by RiboBio. The RT-qPCR detection for miR-125b-5p using TB Green Premix Ex Taq II (Takara), and U6 was used as the normalization control. The 2^−ΔΔCт^ method was used to evaluate gene expression.

### In vitro T-cell culture and stimulation assays

Mouse CD4 T cells and CD4^+^CD25^+^ Treg cells were purified using mouse CD4 (L3T4) microbeads and CD4^+^CD25^+^ Regulatory T Cell Isolation Kit (Cat# 130091041, Miltenyi Biotec), respectively. The purified cells were cultured in a 96-well round-bottom plate and treated with exosomal miR-125b-5p derived from MSC cells (MSC-EXO-miR-125b-5p). For in vitro assays of the percentage and suppression of proliferation by Tregs as described previously [6]. Moreover, to examine the uptake of exosomes by T cells, the exosomes were labeled by DiD dye and cocultured with CD4 T cells for 6–8 h, then photographed with confocal laser scanning microscope. In addition, mouse CD8 T cells were purified using the CD8a^+^ T cell isolation kit, and anti-CD3/anti-CD28 were used to stimulate T cell activation.

### Intracellular staining and flow cytometry

Cells were blocked non-specific FcR and incubated with diluted antibodies according to the manual instructions. For intracellular staining of IFNγ, lymphocytes were stimulated by the Cell Stimulation Cocktail (500×), including PMA and ionomycin, then the cells were fixed in fixation/permeabilization buffer, then resuspended in diluted fixation/permeabilization buffer. The intracellular staining of FoxP3 was similar, but with no cocktail stimulation. All cells were acquired using the BD Fortessa cytometer (BD Biosciences), and data was analyzed by FlowJo software (version 10.8). FACS gating was based on the live cells by applying a LIVE/DEAD Fixable Dead Cell Stain kit.

### The generation and treatment of mouse colon cancer model

C57BL/6J (female, 8 ~ 12 weeks old) were subcutaneously injected with 0.1 ml PBS containing 5 × 10^5^ cells of MC38 in the right flank, when tumor size reached 50 ~ 100 mm^3^, the mice were randomly divided into four groups (*n* = 5 mice/group). The tumor-bearing mice were administrated MSC-EXO-miR-125b-5p (100 µg/mice) via intravenous injection into the tail veil, and intraperitoneal (i.p.) injection of anti-PD-1 antibody (100 µg/mice) alone or in combination twice a week for two weeks. The control group was injected with MSC-EXO-miR-NC (100 µg/mice) via intravenous injection in conjunction with IgG (i.p., 100 µg/mice) for the same duration. The tumor size, body weight, and tumor-bearing mice survival were monitored every three days. The tumor size was calculated by the formula: (length × width^2^)/2. According to the conventional procedure, we did not utilize any particular chemical agents as the anesthetic for experimental mice in this study. However, the mice were euthanized with a certain amount of carbon dioxide in an induction chamber when they reached an endpoint including tumor size > 2000 mm^3^, the mice had a 20% weight loss or exhibited large necrotic areas on the skin. All animal experiments were performed two times independently.

### Tumor tissue processing and inflammatory cytokine assay

After the treatment of MC38 tumor model for a total of three doses, the peripheral blood was harvested from tumor-bearing mice for the isolation of serum. Meanwhile, the tumors were excised and digested by using 0.1 mg/mL DNase I and 100 U/mL collagenase IV at 37℃ for 45 min. The cell suspensions were passed through 70-µm strainers and washed three times by PBS, then stained with live/dead dye before being fixed. For tumor-infiltrating immune cell profiling, the appropriate antibodies were stained before flow cytometry. Moreover, the mouse inflammatory cytokines were analyzed using the mouse inflammation kit by BD cytometric bead array, according to the manufacture instructions.

The work has been reported in line with the ARRIVE guidelines 2.0.

### Statistical analysis

All in vitro and in vivo experiments were performed at least two or three times. Comparisons between two groups were analyzed by student’s t test, and the multiple groups comparison were analyzed by one-way ANOVA test using GraphPad Prism 8.3.0 (GraphPad, San Diego, CA, USA). When conducting these parametric tests, the Shapiro-Wilk test was performed to assess normality. For samples with heteroscedasticity, the Mann-Whitney U test and Kruskal-Wallis test were utilized to evaluate differences. The summarized data were represented as mean ± standard deviation (SD) from at least two independent experiments. *P* value ≤ 0.05 determines a statistically significant difference.

## Results and discussion

### MSC-derived exosomes inhibit FoxP3^+^ Tregs by delivering miR-125b-5p

**Fig. 1:**
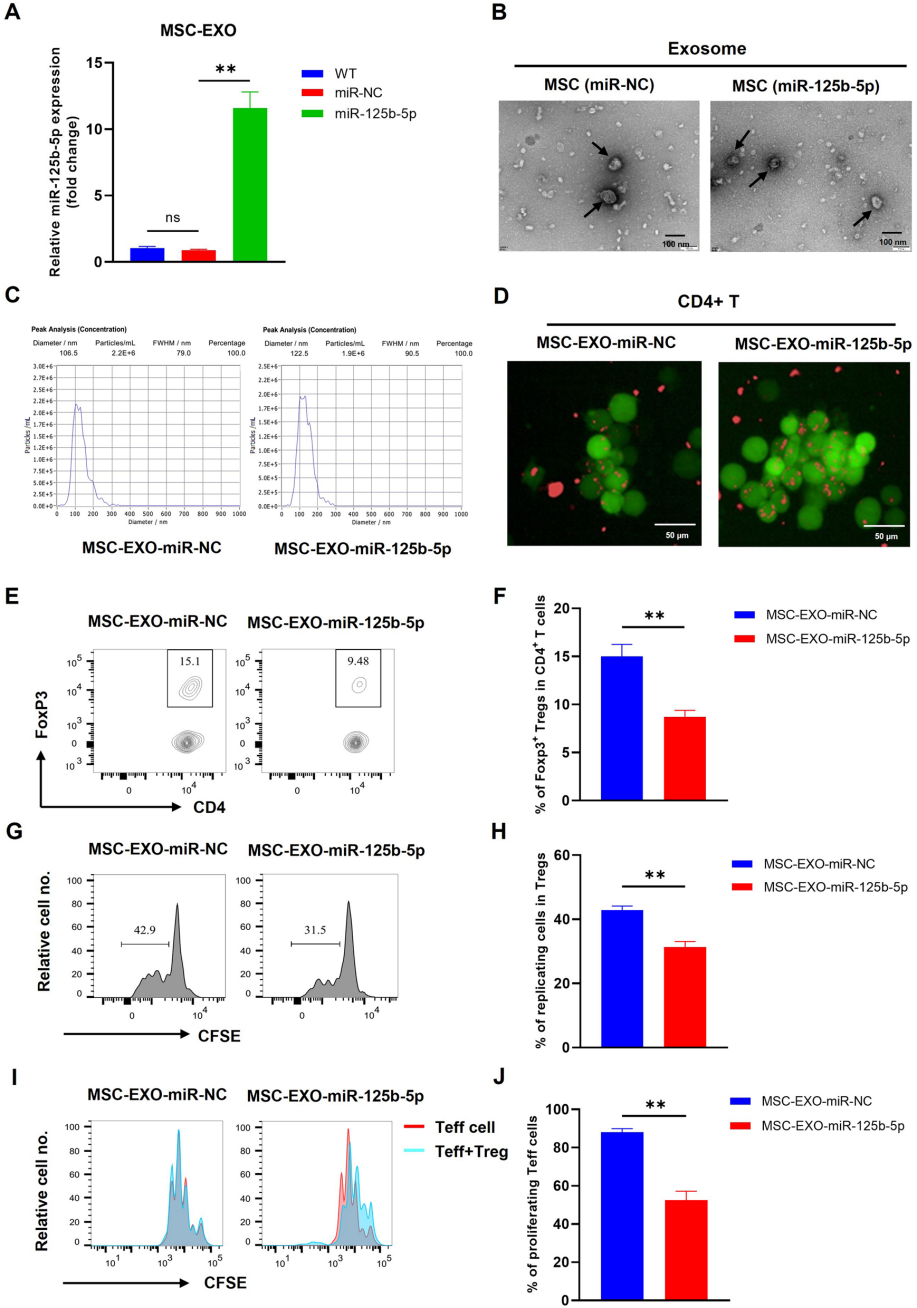
Exosomal miR-125b-5p inhibited the proportion, proliferation, and suppressive function of Tregs. miR-125b-5p-expressing mesenchymal stem cell (MSC) was generated by infection of a lentiviral vector expressing miR-125b-5p, then the exosomes were isolated, and utilized for treating MACS-purified CD4^+^ T or CD4^+^CD25^+^ Treg cells. T cells were cultured in the presence of 10 ng/ml IL-2 and TNF for maintaining their survival and expansion, then treated with 100 µg/ml MSC-derived exosomes (MSC-EXO-miR-NC, or MSC-EXO-miR-125b-5p) for three days. (**A**) The miR-125b-5p relative expression was quantified by RT-qPCR in exosomes derived from MSC (WT, or LV-miR-NC, or LV-miR-125b-5p). (**B**) TEM images of MSC-derived exosomes. (**C**) The average size of MSC-derived exosomes by NTA analysis. (**D**) The confocal microscopy imaging showed that MSSC-EXO could be encapsulated by CD4^+^ T cells. Representative flow cytometric plots (**E**) and summary (**F**) of the percentage of CD4^+^Foxp3^+^ Tregs in CD4^+^ T cells were shown. (**G**) Typical FACS analysis (**G**) and summary (**H**) of Tregs proliferation, as shown by dilution of CFSE expression (gated on Foxp3^+^ Tregs). Representative FACS histogram (**I**) and summary (**J**) of proliferating Teffs cocultured with Tregs that treated with exosomes (Teff: Treg = 1:1). Summarized data (mean ± SD) shown were representatives of three independent experiments with similar results. By comparison with the control exosomes (MSC-EXO-miR-NC), *, *P* < 0.05, ** *P* < 0.01

To verify the potential role of MSC-EXO on the reduction of Tregs by transferring miR-125b-5p, we first generated miR-125b-5p stably expressing cells, and purified the exosomes from MSC (WT, LV-miR-NC, or LV-miR-125b-5p). RT-qPCR analysis showed that the expression of miR-125b-5p was significantly higher in the exosomes derived from miR-125b-5p-expressing MSC compared to control exosomes (Fig. 1A, *p* < 0.01), indicating that miR-125b-5p could be delivered by the MSC-EXO. Furthermore, consistent with previous report of the characteristics of exosomes [14]. We confirmed that the average size distribution of exosomes was between 30~150 nm through nanoparticle tracking analysis (Fig. 1B), and the morphology of the exosomes was characterized using electron microscopy (Fig. 1C). Then, we purified CD4^+^ T cells and cocultured them with 100 µg/ml DiD-labeled MSC-EXO-miR-125b-5p or MSC-EXO-miR-NC, and verified that the exosomes could be encapsulated by CD4^+^ T cells (Fig. 1D). To examine the effect of exosomal miR-125b-5p on Tregs, we found that the proportion of FoxP3^+^ Tregs was reduced by ~ 40% in CD4^+^ T cells (Fig. 1E, F, *p* < 0.01), and the proliferation of FoxP3^+^ Tregs was decreased by ~ 30% after being treated with MSC-EXO-miR-125b-5p (Fig. 1G, H, *p* < 0.01). Moreover, the suppressive effect of Tregs on CD4^+^CD25^−^ effector T (Teff) cells was also significantly reduced by exosome-delivered miR-125b-5p (Fig. 1I, J, *p* < 0.01). Since TNFR2 is a target of miR-125b-5p, the results showed that expression of TNFR2 was also decreased by treatment with MSC-EXO-miR-125b-5p in Tregs (Fig. S1A-B, *p* < 0.05), and meanwhile, the relative co-inhibitory markers, including CTLA-4, PD-L1, and LAG-3, by Tregs were all decreased as well (Fig. S1C, *p* < 0.05). In contrast, the exosome delivered miR-125b-5p had no significant effects on the viability and proliferation of CD8 T cells when activated by anti-CD3/anti-CD28 antibodies (Fig. S2, *p* > 0.05). Thus, our results indicated that the MSC-derived exosomes preferentially inhibit the proliferation and suppressive phenotype of Treg cells by delivering active miR-125b-5p.

**Fig. 2:**
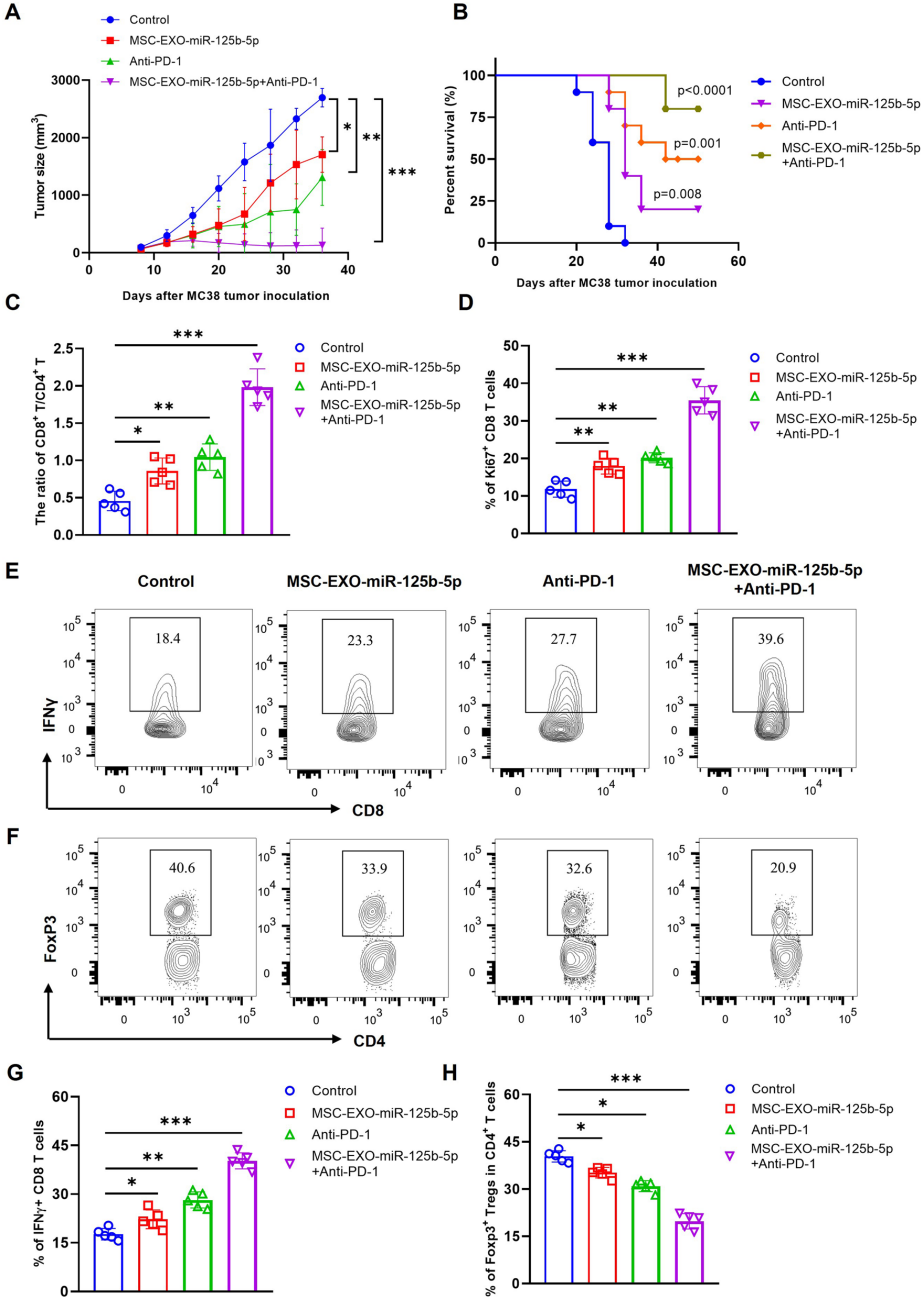
Exosomal miR-125b-5p sensitizes anti-PD-1 therapy in murine colon tumor model by increasing tumor-infiltrating CD8 CTLs and reducing Tregs. C57BL/6J mice were subcutaneous (s.c.) inoculated with MC38 tumor cells to the right flank of each mouse on day 1, then randomly divided into four groups. When tumor size reached ~100 mm^3^, mice were treated by injection of MSC-EXO-miR-125b-5p into tail veil with or without anti-PD-1 antibody (i.p., 100 µg/mouse) every three days for two weeks. The control group was defined by administered MSC-EXO-miR-NC and IgG. The immune cells in tumor tissue were examined by gating on live cells with flow cytometry. (**A**) The tumor growth curve was plotted from each group (*n* = 10). (**B**) Survival curve (Kaplan-Meier plotter). Summary of the ratio of CD8 versus CD4 T cells (**C**), and Ki67 expression by CD8 T cells (**D**) were shown. Typical FACS plot (**E**) and summary (G) of IFNγ-expressing CD8 T cells were shown. Representative flow cytometric plot (**F**) and summary (**H**) of the percentage of Foxp3^+^ Tregs in CD4^+^ T cells in mouse tumor tissues. For typical FCM plots, the number indicated the proportion of gated cells. The summarized data shown were represented as mean ± SD, *n* = 5 mice of each group. The results data in A and B were pooled from two independent experiments (*n* = 10). By comparison with the control group, *, *P* < 0.05, ** *P* < 0.01, *** *P* < 0.001

### Exosomal miR-125b-5p enhances the efficacy of anti-PD-1 therapy by modulating tumor microenvironment in murine colon tumor model

To determine the in vivo effect of exosomal miR-125b-5p on tumor growth, MC38 tumor bearing mice were treated with MSC-EXO-miR-125b-5p (100 µg/mice) for up to four doses (twice/week), when tumor size reached at 100mm^3^ from d8~d10. However, the results showed that only 2 out of 10 mice (~20%) received a complete regression after the treatment of MSC-EXO-miR-125b-5p, and with tumor growth moderately inhibited (Fig. S3, *p* < 0.05). Thus, to test the idea of exosomal miR-125b-5p may enhance the efficacy of anti-PD-1 therapy in cancer treatment, we further treated MC38 tumor model by the combination of MSC-EXO-miR-125b-5p and anti-PD-1 antibody. Interestingly, the results showed that the single treatment of anti-PD-1 (100 µg/mice) could result in a cure rate of~30% (3/10) in MC38 tumor-bearing mice, similarly to other reported before [15]. while the combined treatment of MSC-EXO-miR-125b-5p and anti-PD-1 therapy resulted in~80% (8/10) mice reached complete regression, with tumor growth significantly inhibited (Fig. 2A, *p* < 0.05). As predicted, the long-term survival of tumor-bearing mice was markedly improved in the combined treatment group as well (Fig. 2B). To analysis the tumor immune environment, we found a notable increase of ratio of CD8/CD4 T cells with high levels of proliferating CD8 T cells in tumor tissue after the combination of MSC-EXO-miR-125b-5p with anti-PD-1 antibody (Fig. 2C, D, *p* < 0.05). Meanwhile, the proliferation and IFNγ expression by CD8 T cells was increased significantly as well (Fig. 2E, G, *p* < 0.05). On the contrary, the proportion of FoxP3^+^ Tregs was markedly decreased (Fig. 2F, H, *p* < 0.05). Moreover, we also examined the circulating inflammatory cytokine levels in tumor bearing mice, the results showed the decreased levels of IL-6, TNF, and IL-12, but increased IFN-γ after MSC-EXO-miR-125b-5p with or without anti-PD-1 treatment (Fig. S4, *p* < 0.05). Additionally, the potential effect of exosomal miR-125b-5p on immune cells in other compartments, such as monocytes, and B cells, present no obvious change (data not shown). Our results suggest that exosomal miR-125b-5p derived from MSC cells may enhance the efficacy of anti-PD-1 therapy by eliminating the immunosuppressive tumor-infiltrating Tregs and mobilization of CD8 CTLs.

Previously, it has been reported that a high level of miR-125b-5p expression in tumor tissue is associated with increased immune cell infiltration [16]. Ectopic expression of certain miRNAs plays a critical role in the regulation of PD-1/PD-L1 immune checkpoint [17, 18], and some have been confirmed to predict the response to anti-PD-1 therapy [19]. However, whether miR-125b-5p can enhance ICIs therapy remains incompletely understood. To date, although some miRNAs-based therapeutics have been approved for clinical trials, such as a miR-34 mimic for cancer treatment [20] and anti-miR-122 for hepatitis treatment [21], miRNAs are generally small, water-soluble, and easily degraded, making systemic delivery in cancer treatment challenging [22]. Exosomes are recognized as vehicles for signal transduction and play a significant role in cancer progression by modulating intercellular communication, including regulating immune responses, reprogramming the tumor microenvironment, and conferring drug resistance to tumor cells [23–25]. Currently, there is a growing body of evidence suggesting that miR-125b-5p can be delivered by specific exosomes. For example, melanoma-derived exosomal miR-125b-5p has been shown to induce tumor-associated macrophages to adopt a tumor-promoting phenotype [26], while exosome-containing miR-125b possessed anti-metastatic properties in hepatocellular carcinoma (HCC) [27].

To date, exosomes have demonstrated significant advantages as an ideal drug delivery system, particularly those derived from MSCs. Numerous studies have indicated that MSC-derived exosomes (MSC-EXO) can effectively enhance the therapeutic efficacy of chemotherapeutic agents and radiotherapy in cancer treatment by transferring synthetic miRNAs [28, 29]. Thus, in this study, we posit that MSC-EXO could be utilized for the delivery of active miR-125b-5p. Initially, we confirmed that overexpression of miR-125b-5p in MSC results in the secretion of exosomes enriched with miR-125b-5p. Notably, in vitro analyses revealed that exosomal miR-125b-5p derived from MSC can be transferred to T cells, leading to a decrease in the number of FoxP3^+^ Tregs within the CD4 T cell population and inhibiting Treg proliferation, while having minimal effects on CD8 T cells. These findings suggest that exosome-mediated delivery of miR-125b-5p selectively targets immunosuppressive Tregs. Given the limited number of studies examining the correlation of miR-125b-5p with ICIs therapy, in this study, we first verified that the administration of exosomal miR-125b-5p alone in tumor-bearing mice led to partial tumor regression. However, when combined with anti-PD-1 therapy, the efficacy was significantly enhanced by two- to three- times, achieving complete tumor regression in approximately 80% of the mice. More importantly, within the tumor microenvironment, there was a significant reduction in tumor-infiltrating Tregs and a marked increase in CD8 CTLs, particularly in the combined treatment of MSC-EXO-miR-125b-5p and anti-PD-1 antibodies. Consequently, this study provides evidence that exosomal miR-125b-5p derived from MSC effectively enhances the sensitivity of cancer cells to immunotherapy.

At present, research has shown that MSC-derived exosomes mediate the primary therapeutic effects of MSCs in various diseases, thereby establishing them as a promising cell-free therapeutic strategy in cancer treatment [30]. However, the clinical translation of MSC-derived exosomes is accompanied by both opportunities and challenges. Key issues that require immediate attention include improving the stability of exosomes, elucidating their mechanisms of action, and reducing potential off-target effects. Given that multiple factors can affect the stability of exosomes, numerous studies have been dedicated to enhancing their structural integrity and preserving their internal bioactive molecules through targeted modifications, and the incorporation of nanomaterials [31]. Moreover, the biodistribution of exosomes within the body is modulated by various factors, including the route of administration and the intrinsic physical and chemical properties of the exosomes [32]. Nevertheless, MSC-derived exosomes still hold significant promise for clinical use. Thus, by thoroughly studying how exosomes interact with different cell types and identifying crucial factors and targets for their therapeutic effects, we can improve therapeutic designs for more precise and effective outcomes.

## Conclusions

In conclusion, our findings indicate that MSC-derived exosomal miR-125b-5p enhances the efficacy of anti-PD-1 therapy in cancer treatment, thereby providing robust evidence for the translational research of miRNAs in future therapeutic strategies. The ability of miR-125b-5p to enhance the effectiveness of tumor immunotherapeutic agents holds significant potential for clinical application.

## Electronic supplementary material

Below is the link to the electronic supplementary material.

Supplementary Material 1

## Data Availability

The data used in this article is available from the corresponding author upon appropriate request.

## Abbreviations

CR: Complete response
CTLs: Cytotoxic T lymphocytes
EMT: Epithelial-mesenchymal transition
FoxP3: Forkhead box protein P3
HCC: Hepatocellular carcinoma
ICIs: Immune checkpoint inhibitors
MSC: Mesenchymal stem cell
MSC-EXO: Mesenchymal stem cell derived exosome
NTA: nanoparticle tracking analysis
PD-1: Programmed cell death receptor 1
PD-L1: Programmed cell death ligand 1
RT-qPCR: Real-time fluorescence quantitative PCR
TEM: Transmission electron microscopy
TME: Tumor microenvironment
TNFR2: Tumor necrosis factor receptor 2
Tregs: Regulatory T cells
