# Supplementary material for: Exosomal miR-125b-5p derived from mesenchymal stromal/stem cell enhances anti-PD-1 therapy in mouse colon cancer model
Source: Stem Cell Res Ther. 2025 Mar 5;16:112. doi: 10.1186/s13287-025-04227-3 (PMC11881248; doi:10.1186/s13287-025-04227-3)
Supplement: Supplementary file 1 — Supplementary Material 1 [file 13287_2025_4227_MOESM1_ESM.docx]

Supplementary Materials for

**Exosomal miR-125b-5p derived from mesenchymal stromal/stem cell enhances anti-PD-1 therapy in mouse colon cancer model**

Mengmeng Jiang, Jia Liu, Shengquan Hu, Xueqin Yan, Yongkai Cao, Zhengzhi Wu^*^

* Corresponding author:

Zhengzhi Wu, Foreign academician of the Ukrainian National Academy of the National Academy of Engineering Sciences, Shenzhen, China.

Email: szwzz001@email.szu.edu.cn

**The PDF file includes:**

Figure S1. MSC-derived exosomal miR-125b-5p inhibited the expression of typical surface markers of Treg cells.

Figure S2. Effect of MSC-derived exosomal miR-125b-5p on the proliferation of CD8 T cells.

Figure S3. MSC-derived exosomal miR-125b-5p inhibited the tumor growth in MC38 tumor model.

Figure S4. The expression levels of inflammatory cytokines in tumor model after the treatment of exosomal miR-125b-5p and anti-PD-1.

**
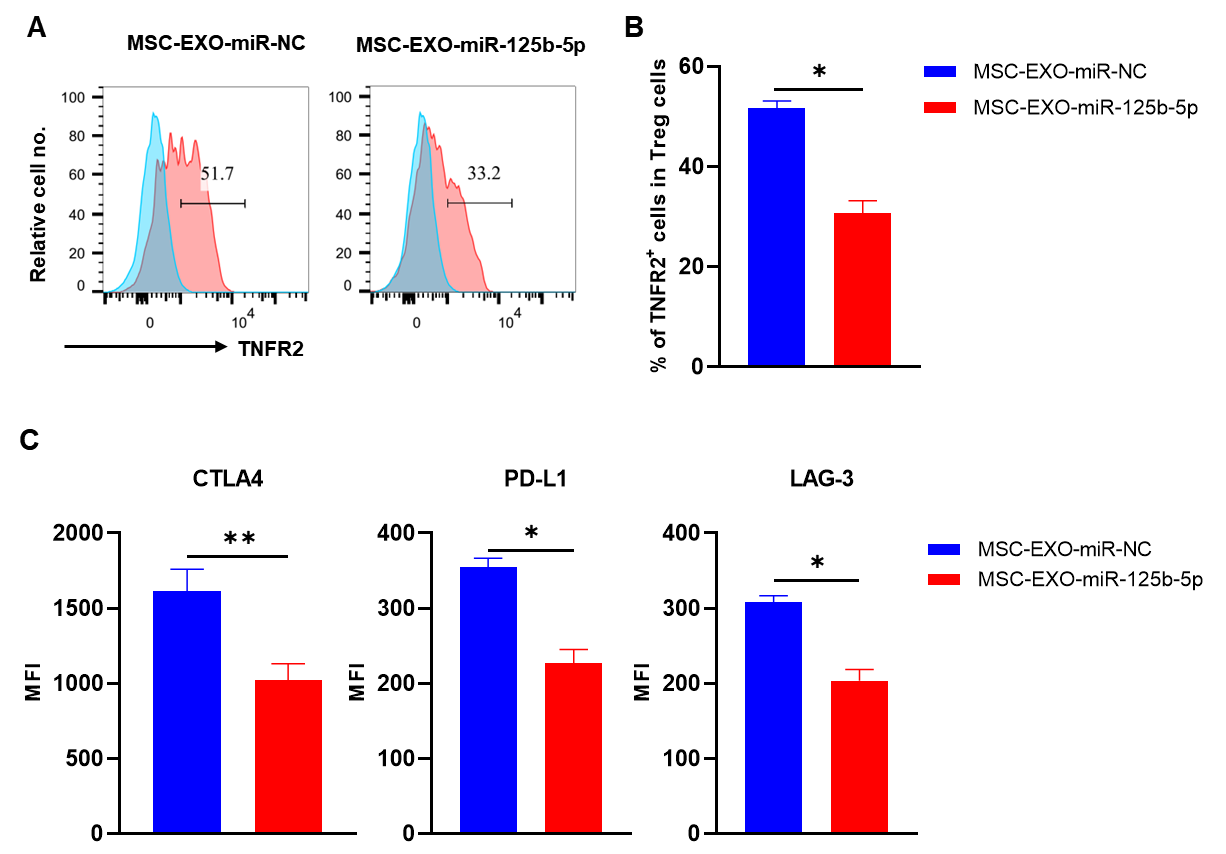
**

**Fig. S1. MSC-derived exosomal miR-125b-5p inhibits the expression of typical surface markers of Tregs.** MACS-purified CD4^+^ T cells from pooled lymph nodes and spleen of mice were cultured in the presence of 10 ng/ml IL-2 and TNF, then treated with 100 μg/ml MSC-derived exosomes (miR-NC, or miR-125b-5p) for three days. Representative FACS histogram (A) and summary (B) of TNFR2 expression by Tregs. (C) The mean fluorescence intensity (MFI) of CTLA-4, PD-L1, and LAG-3 expression by Tregs. For typical FCM plots, the number indicated the proportion of gated cells. Summarized data (mean ± SD) shown were representatives of three independent experiments with similar results. By comparison with the control exosomes (MSC-EXO-miR-NC), *, *P*<0.05, ** *P*<0.01.


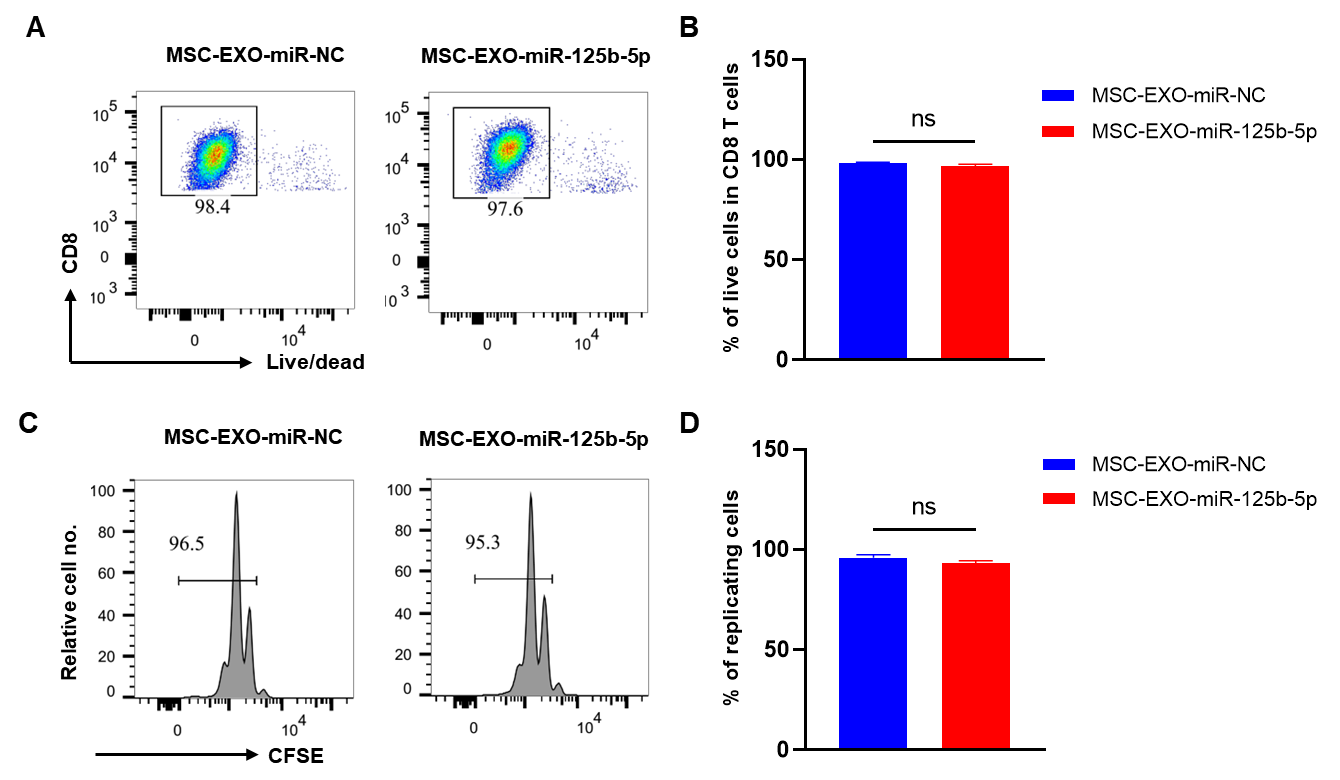


**Fig. S2. Effect of MSC-derived exosomal miR-125b-5p on the viability and proliferation of CD8 T cells.**

MACS-purified CD8 T cells from pooled lymph nodes and spleen mice were cultured in the presence of 10 ng/ml IL-2 and anti-CD3/CD28 antibodies, then treated with 100 μg/ml MSC-derived exosomes (miR-NC, miR-125b-5p) for three days. The cell viability of CD8 T cells were assessed by gated on live cells. Representative flow cytometric graph (A) and summary (B) of live cells in CD8 T cells. Typical FACS analysis (C) and summary (D) of cell proliferation in CD8 T cells, as shown by dilution of CFSE expression. Representative FACS histogram was from three independent experiments with similar results. Summarized data were represented as mean ± SD from three independent experiments. By comparison with control exosomes (MSC-EXO-miR-NC), ns, no significance.


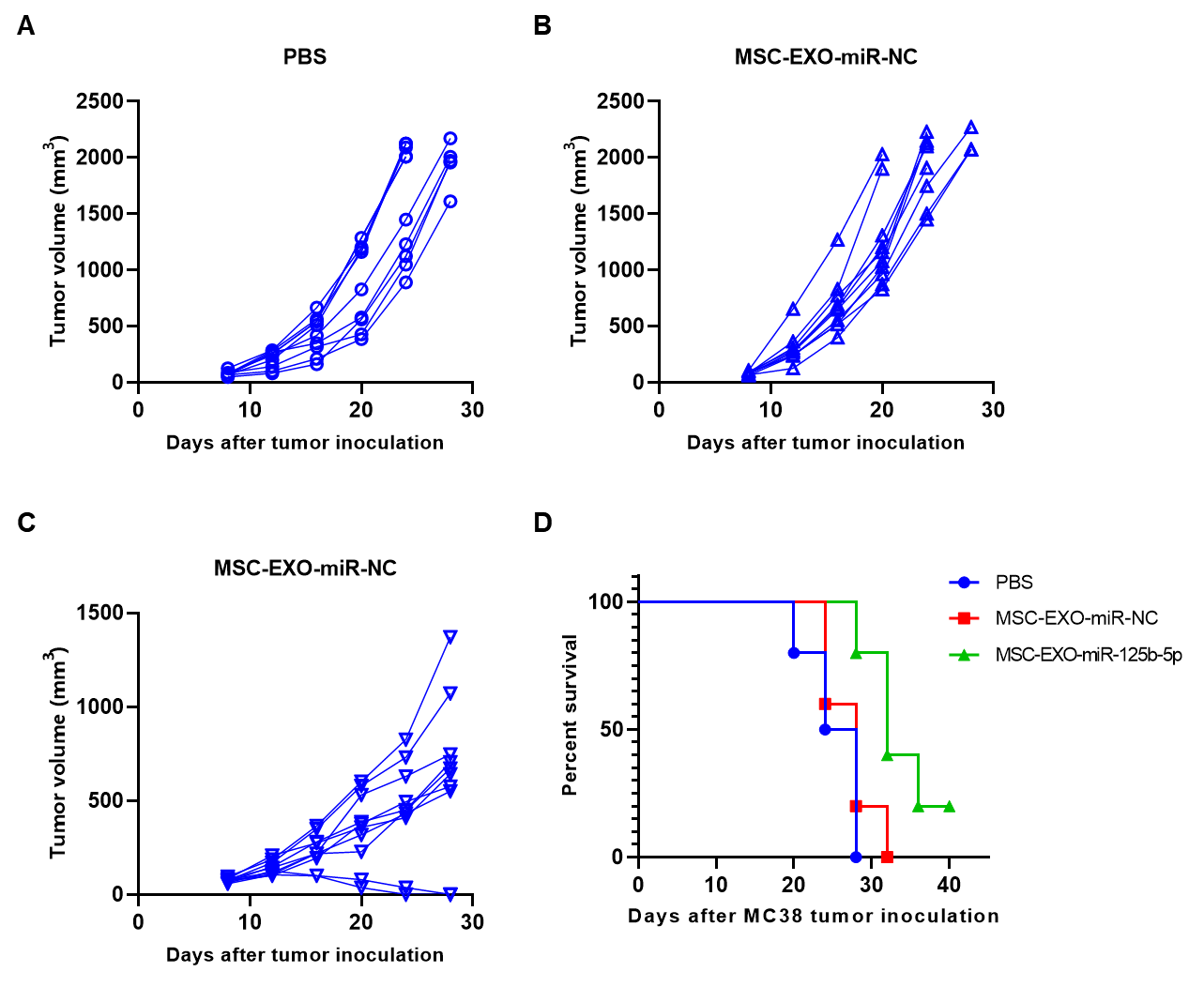


**Fig. S3.** **MSC-derived exosomal miR-125b-5p inhibited the tumor growth in MC38 tumor model.** C57BL/6J mice were subcutaneous (s.c.) inoculated with MC38 tumor cells on the right flank on day 1. When tumor size was reached at 100mm^3^, the tumor bearing mice will intraperitoneal injected with MSC-EXO-miR-125b-5p (100μg/mouse) for up to four doses. The tumor growth curve was plotted from each mouse treated with PBS (A), or MSC-EXO-miR-NC (B), or MSC-EXO-miR-125b-5p (C). (D) Survival curve (Kaplan-Meier plotter). The results data were pooled from two independent experiments (n=10).


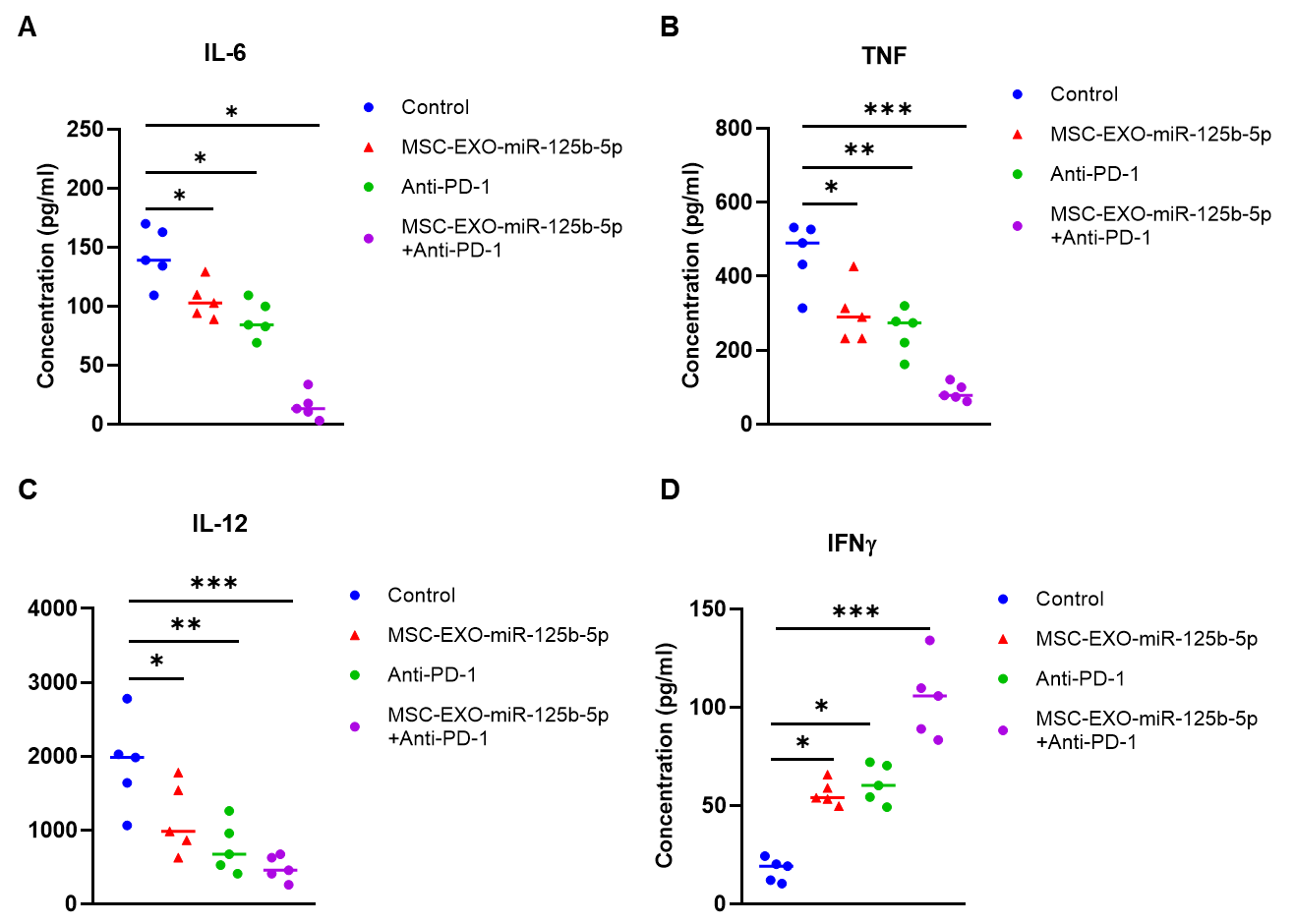


**Fig. S4. The expression levels of inflammatory cytokines in tumor model after the treatment of exosomal miR-125b-5p and anti-PD-1.** C57BL/6J mice were subcutaneous (s.c.) inoculated with MC38 tumor cells to the right flank of each mouse on day 1, then randomly divided into four groups. When tumor size reached 100 mm^3^, mice were intraperitoneal (i.p.) injected with MSC-EXO-miR-125b-5p with or without anti-PD-1 antibody (100 μg/mouse) every three days for two weeks. The control group was defined by administered MSC-EXO-miR-NC and IgG. After two times therapy, the tumor bearing mice were sacrificed, and the peripheral blood was selected for ELISA assay. The serum levels of inflammatory cytokine IL-6 (A), TNF (B), IL-12 (C), and IFN-γ (D) were shown. The summarized data shown were represented as mean ± SD, n=5 mice of each group. The results data representatives of two separate experiments. By comparison with the control group, *, *P*<0.05, ** *P*<0.01, *** *P*<0.001.
